# Protein retention on plasma-treated hierarchical nanoscale gold-silver platform

**DOI:** 10.1038/srep13379

**Published:** 2015-08-26

**Authors:** Jinghua Fang, Igor Levchenko, Anne Mai-Prochnow, Michael Keidar, Uros Cvelbar, Gregor Filipic, Zhao Jun Han, Kostya (Ken) Ostrikov

**Affiliations:** 1 Plasma Nanoscience Laboratories, Manufacturing Flagship, Commonwealth Scientific and Industrial Research Organisation (CSIRO), P.O. Box 218, Lindfield, NSW 2070, Australia; 2 School of Physics, University of Melbourne, Parkville, VIC, Australia, 3010; 3 School of Chemistry, Physics, and Mechanical Engineering, Queensland University of Technology, Brisbane, QLD 4000, Australia; 4 Department of Mechanical and Aerospace Engineering, The George Washington University, Washington, DC 20052, USA; 5 Jozef Stefan Institute, Dep. of Surface Eng. and Optoelectronics, Jamova 39, 1000 Ljubljana, Slovenia, EU; 6 Plasma Nanoscience, School of Physics, The University of Sydney, Sydney, NSW 2006, Australia

## Abstract

Dense arrays of gold-supported silver nanowires of about 100 nm in diameter grown directly in the channels of nanoporous aluminium oxide membrane were fabricated and tested as a novel platform for the immobilization and retention of BSA proteins in the microbial-protective environments. Additional treatment of the silver nanowires using low-temperature plasmas in the inductively-coupled plasma reactor and an atmospheric-pressure plasma jet have demonstrated that the morphology of the nanowire array can be controlled and the amount of the retained protein may be increased due to the plasma effect. A combination of the neutral gold sublayer with the antimicrobial properties of silver nanowires could significantly enhance the efficiency of the platforms used in various biotechnological processes.

Immobilization of various biologically-active species such as proteins and enzymatic biocatalysts^1^, proteins^2^, biomolecules^3^, living cells^4^, DNA^5^, red blood cells^6^, stem cells^7^, bioethanol-producing bacteria^8^ and others is required for many applications including biofuel cells^9^, food processing^10^ and energy conversion devices^11^, sensors^12^, biosensors^13^, virus detection^14^, microfluidic^15^ and drug delivery devices^16^, fluidised bed bioreactors^17^ and fixed-bed catalytic reactors^18^. As a supporting platform, various structures such as mesoporous carbon beads^19^, nanostructured polymer surfaces^20^, silica/polymer matrices^21^ and nanoporous membranes^22^ with the nanometre-scale morphology for enhancing protein adsorption^23^ are commonly used. Recently, novel hierarchical architectures based on vertical carbon nanotubes^24^,^25^ and vertical carbon nanowalls^26^ were proposed.

Among others, inorganic and metallic platforms based on noble metals are important owing to several useful properties such as very high chemical stability and inertness, absence of carbonous and other contaminations (which is of special importance for biosensing applications^27^ and highly sensitive diagnostics in medicine^28^), as well as high-temperature operation (e.g., for the fast and convenient deactivation and sterilization).

A detailed analysis of the possible combinations of materials (chemical aspect) and structures (physical aspect) attracts attention to the combination of a solid gold base and an array of silver nanowires on the top^29^. Indeed, one-dimensional nanostructures (nanowires, nanofibers, nanocones, etc.) grown on solid supporting sublayers are used in various applications, including nanoelectronics^30^, as well as energy storage and conversion devices^31^. The use of nanostructures and nanoarchitectures made of noble metals such as gold, platinum and silver are of special importance for several medical and biotechnological applications due to a strong antimicrobial activity of silver nanowires^32^ and nanoparticles^33^,^34^, as well as complex nanoarchitectures involving silver and gold nanostructures^35^. Besides, gold is known as a suitable base for immobilizing biological species and biomolecules^36^.

The ability of such structures to capture and retain enzymes and proteins, in particular the enzyme-stabilizing proteins such as bovine serum albumin (BSA) is an important feature of nanoarchitectures based on long one-dimensional nanostructures^37^. The enzymes and any other biologically active species entrapped in such dense arrays of long nanostructures could be under a dual protection: dense structure with the gaps between the nanostructures of less than one μm mechanically protects the entrapped species from the direct contact with bacteria, whereas the chemical activity of silver nanowires is an additional protection by specific silver-bacterial membrane interaction which leads to effective killing of bacteria^38^. Moreover, the nanostructured silver is very effective in the antimicrobial action due to the controlled release of Ag^39^. Such a protection could result in a significant increase of the process productivity due to conserving expensive biologically active species.

Biocatalysts and proteins are usually immobilized on inorganic platforms by the metal-link technique^40^ or other support activation methods^41^, which involve additional chemical treatment. This compromises the major advantages of the ‘pure metallic’ platforms – chemical inertness and simple chemical composition. To avoid this and to demonstrate the possibility to control the retention of proteins *without any chemical treatment*, we have designed, fabricated and tested a novel nano-architectured plasma-activated platform consisting of a 300 nm gold base and a dense array of silver nanowires.

Nanoporous aluminium oxide (Al_2_O_3_, alumina) membrane was used as a template to form the nanowire array with a density high enough to ensure gaps between the nanowires of several hundred nanometers, i.e., much less than the typical size of common enzyme-producing bacteria such as *E. coli* and *B. subtilis*. A special treatment of the system was then used to demonstrate that the ability of the silver nanowire forest to retain proteins *can be controlled* and the characteristics of the hierarchical compound nanowire-based materials could be significantly enhanced. Indeed, considerable improvement was obtained by using low-temperature plasmas in designing various nanostructures^42^. Microwave^43^, magnetron^44^,^45^, arc^46^ and atmospheric-pressure plasma^47^,^48^ discharges are among the most common systems used for the plasma-based nanofabrication. Plasma was also successfully used for the protein immobilization on carbon nanotubes^49^.

In this work, we have used two different types of plasma treatment. Firstly, a cheap and convenient treatment in the low temperature inductively-coupled plasmas (ICP)^50^, to control the morphology of the nanowires was used. Secondly, the atmospheric-pressure plasma jet to activate the surface was implemented for protein attachment. We did not aim at specifically covalent attachment since it can result in the protein damage, loss of the three-dimensional structure (unfolding) and deactivation^51^. The results obtained show that the plasma-treated nanoscale gold-silver platform (NGSP) is capable of retaining BSA protein molecules.

## Results and Discussion

### Process description

**Figure 1:**
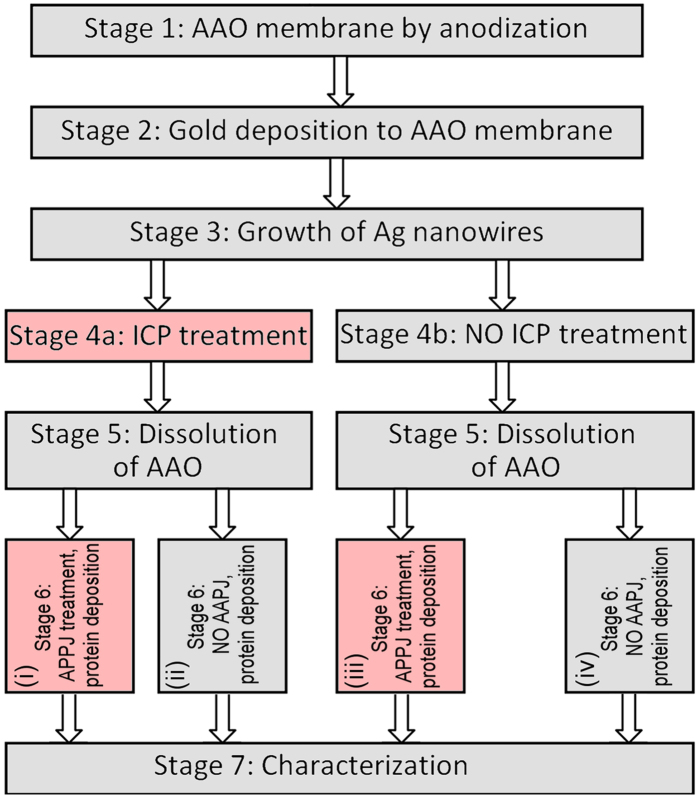
Schematic of the process. The stages where plasmas were used are filled with pink colour. The series of samples with proteins deposited are marked: (i) ICP only treated; (ii) ICP and APPJ treated; (iii) no-ICP, APPJ treated; (iv) no-ICP, no-APPJ treated.

A schematic diagram illustrating the sequence of specific stages in the whole process is shown in Fig. 1. The process was started from the AAO membrane preparation (Stage 1) followed by the gold deposition onto one side of the membrane (Stage 2) and growth of silver nanowires in the membrane pores (Stage 3). After that, several samples were treated using ICP (Stage 4) whereas other samples were kept untreated. Then all samples (ICP treated and non-treated) were subject to AAO dissolution (Stage 5). Finally, part of the ICP treated and non-treated samples were processed using the atmospheric pressure plasma jet (APPJ), and proteins were deposited onto all samples (Stage 6). At the final Stage 7, all samples were characterized as described below. Thus, the four series of the samples with proteins were prepared: (i) ICP only treated; (ii) ICP and APPJ treated; (iii) no-ICP, APPJ treated; (iv) no-ICP, no-APPJ treated. The relevant marks (i, ii, iii, iv) are also shown on Fig. 1.

### Fabrication of Free-Standing Silver Nanowire Arrays on Gold Layer

**Figure 2:**
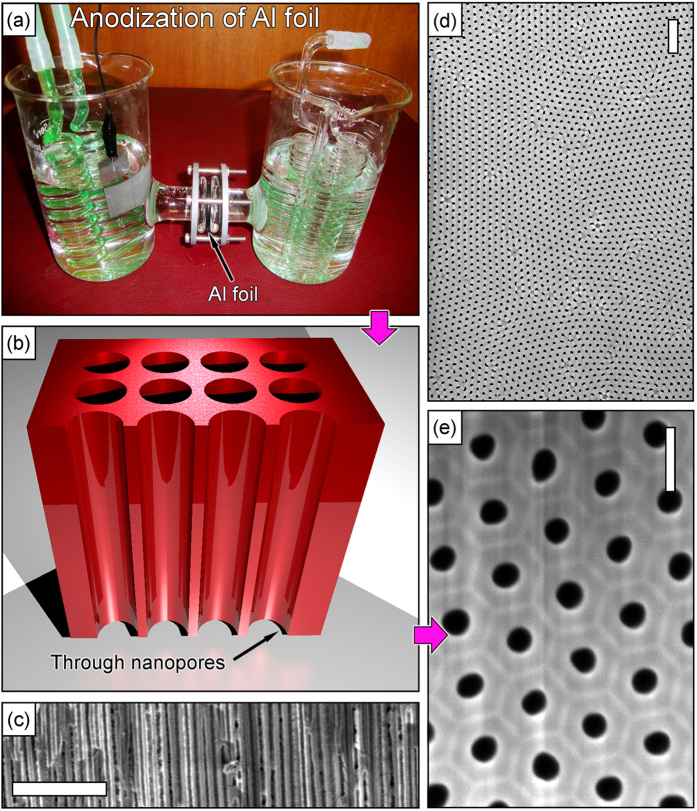
Alumina membrane fabrication and SEM images of the as-prepared membrane. (**a**) Aluminium foil (250 μm thick) is anodized in oxalic acid to produce the nanoporous membrane. (**b**) 3D representation of the membrane structure. The nanochannels are through after additional etching of the membrane bottom in CuCl_2_ + HCl. (c) SEM image of the membrane cross-section. Straight nanochannels with the diameter of about 100 nm are visible. (**d**,**e**) Low- and high-magnification SEM images of the membrane (top view). Scale bars are 1 μm (**c**), 500 nm (**d**) and 100 nm (**e**).

Schematic of the AAO membrane fabrication process (Stage 1, Fig. 1) is shown in Fig. 2a. Figure 2b shows the 3D visualization of the as-prepared AAO membrane. SEM images of the prepared membranes are shown in Fig. 2c (cross-section illustrating the straight channel) and Fig. 2d,e (low- and high-magnification SEM images of the upper surface of the membrane). The highly ordered pores are open from the both sides of the membrane, an average pore diameter is 60–70 nm, and the distance between the pores is about 100 nm. The prepared nanoporous membranes (external diameter of about 20 mm) were then coated with gold from one side (Stage 2, Fig. 1).

**Figure 3:**
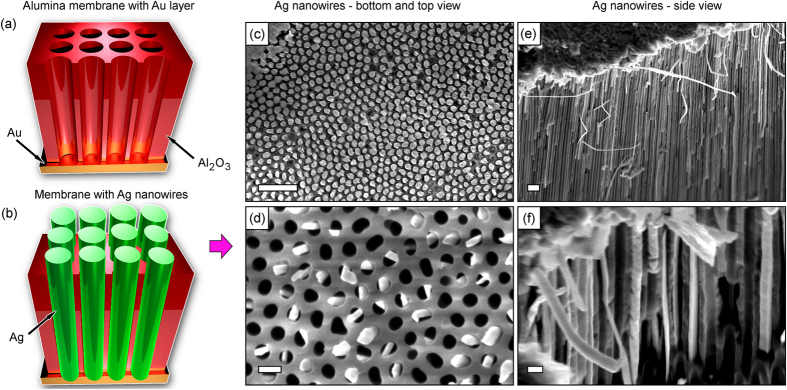
Schematic and SEM photos. Schematic of gold (300 nm) layer deposition onto the bottom of membrane (**a**) and growth of silver nanowires in the channels of the membrane (**b**). (**c**,**d**) SEM images of the bottom and top surfaces of the alumina membrane with silver nanowires in the channels, respectively. All channels are filled with Ag nanowires from the bottom side (**c**), whereas not all channels are filled on the upper side (**d**). (**e**,**f**) Ag nanowires in the membrane channels, cross-sectional side view. Scale bars are 500 nm for the upper row (**c**,**e**) and 100 nm for the lower row (**d**,**f**).

Figure 3a,b show the 3D visualization of the AAO membrane with the gold layer and array of silver nanowires in the channels of the membrane (Stage 3, Fig. 1), respectively. SEM images of the bottom (Fig. 3c, after gold layer removal) and top (Fig. 3d) surfaces of the membrane show that all channels are filled with Ag nanowires from the bottom side, whereas not all channels are filled on the upper side. Silver nanowires in the membrane channels (cross-sectional side view) are shown in Fig. 3e,f.

All prepared membranes with silver nanowire arrays were then divided into the two groups. The first group membranes were mounted on the electrode and treated with the ICP plasma for 2 min in Ar at 750 W discharge power (frequency 13.56 MHz) for 2 min (Stage 4a, Fig. 1). The second (reference) group did not undergo any additional treatment (Stage 4b, Fig. 1). More details on the ICP plasma treatment and experimental setup can be found elsewhere^50^.

**Figure 4:**
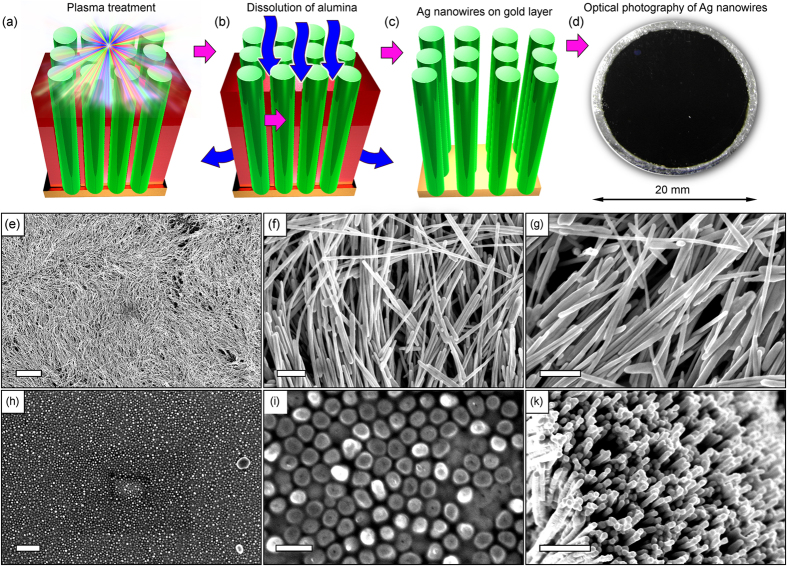
Schematic of the array of silver nanowire fabrication and SEM images of the nanowires on membrane pores. (**a**–**c**) ICP treatment of the nanowires, dissolution of alumina in H_3_PO_4_, and ready array of nanowires on gold layer. (**d**) Optical photography of the Ag nanowires. The reflectivity index is close to zero. (**e**–**g**) Low- and high-magnification SEM images of silver nanowires grown in alumina membrane, after dissolution of the membrane in 5% H_3_PO_4_ acid, without the ICP treatment. The length of nanowires reached several tens of μm. Scale bars are 10 μm (**e**) and 500 nm (**f**,**g**). (**h**–**k**) Low- and high-magnification SEM images of silver nanowires after dissolution of the membrane in H_3_PO_4_ acid and the ICP treatment. The nanowires are much shorter after plasma treatment, as seen from comparison on panels (**g**,**k**). Scale bars are 1 μm (**h**) and 500 nm (**i**,**k**).

Figure 4 illustrates the further processing of the alumina membrane with Ag nanowires and fabrication of the nanowire arrays on the supporting gold layers. After the ICP treatment of the first group (Fig. 4a) all the samples from the group 1 (ICP-treated) and group 2 (non-treated) were immersed into 5% H_3_PO_4_ solution for several hours to dissolve the alumina membrane (Fig. 4b) and leave free standing silver nanowire arrays on the gold supporting film (Fig. 4c). Optical photography of the array of Ag nanowires on the gold layer is shown in Fig. 4d. The reflectivity index of this system is close to zero (the dense array of nanowires absorbs nearly all the incident light).

Figure 4(e–g) are the low- and high-resolution SEM images of the silver nanowires after dissolution of the membrane without the ICP treatment. The length of the nanowires reached several tens of μm. In contrast, the array of nanowires produced by dissolving the membrane after the ICP plasma treatment (Fig. 4h–k) demonstrates shorter nanowires with the lengths reaching only several μm. The silver nanowires were cut by the strong ion flux extracted from the dense plasma (note that the discharge power reached 750 W in our process, this resulted in the plasma density up to 10^18^ m^−3^ 52). Importantly, alumina is an insulator whereas silver is a very good conductor. As a result, the flux of ions extracted from the plasma was concentrated on nanowires which were under negative electric potential during the process (the samples were installed on the table with a bias of about 100 V), and hence, the nanowires were intensively heated and sputtered^53^. More SEM images can be found in the Supplementary Information Fig. S1.

### Protein Interaction with Silver Nanowire Arrays

The samples treated with the atmospheric-pressure plasma jet (APPJ) before protein incubation were installed at the processing table directly under the glass tube, and the plasma was ignited by the 40 W discharge for 2 min. More information about the APPJ processing can be found elsewhere^54^,^55^, as well as in the Methods section of this article.

**Figure 5:**
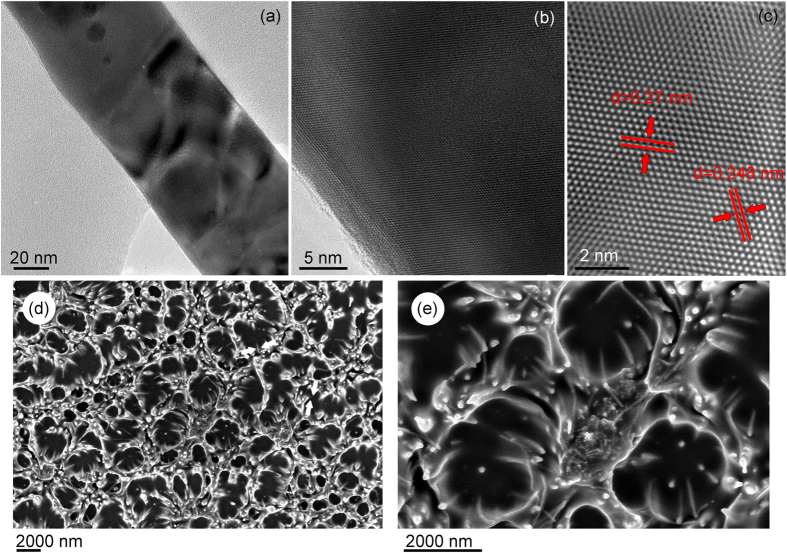
TEM and SEM images of the nanowires and protein-incubated arrays. Low (**a**) and high (**b**,**c**) resolution TEM images of the Ag nanowires. (**d**,**e**) SEM images of the samples after plasma jet treatment and protein deposition to the nanowire array.

The samples were examined by using field-emission scanning electron microscopy (FE-SEM, type Zeiss Auriga) operated at electron beam energy of 1–5 keV with an InLens secondary electron detector. To additionally characterize the fabricated silver nanowires, the structure was studied by the transmission electron microscopy (TEM) technique using the transmission electron microscope JEOL 2100 operated at electron beam energy of 200 keV (Fig. 5a–c). As it follows from the images, the nanowires have polycrystalline structure which is advantageous for many applications including biological and medical, since this structure possesses the active crystal edges. On the other hand, the crystals composing the nanowires are quite large, up to several tens of nm.

For the experiments on trapping and retention of proteins, Bovine Serum Albumin (BSA) was used. This protein is cheap and commonly available, and is usually used as a reference in biochemical and medical experiments, including Enzyme-Linked Immunosorbent Assay (ELISA). BSA has a molecular weight of 66.5 kDa, 583 amino acid residues, with the typical size of about 5 × 15 nm. Examination of the SEM images of silver nanowires (Figs 3 and 4) shows than the nanowire arrays are permeable for BSA molecules (the gaps between nanowires are larger that the BSA molecule size).

In the trapping and retention experiments we used 0.5% BSA solution in water and phosphate buffered saline (PBS). Specifically, we have prepared several samples for studying protein interaction with the silver nanowires. Samples of series 1 were arrays of free-standing silver nanowires on a gold layer, prepared without ICP post-treatment. Samples of series 2 were the arrays of free-standing silver nanowires on gold after the ICP plasma treatment. The samples of both series were incubated in the water and PBS solution of BSA proteins (overnight in 5 μl of the 0.5% BSA solution, following by washing in PBS and water and drying for 5 hours), with and without treatment by the atmospheric-pressure plasma jet (Helium flux, for 2 min at 40 W discharge power). Finally, control samples were made by dropping 5 μl of 0.5% BSA solution onto SiO_2_ (i.e. standard Si wafer covered with a native silica layer of about 500 nm), also dried for 5 hours. The ready samples were investigated using the Raman and XPS techniques which are standard characterizations for detection of the surface-absorbed proteins^56^,^57^. SEM images of the arrays after protein treatment are also shown in Fig. 5(d,e).

**Figure 6:**
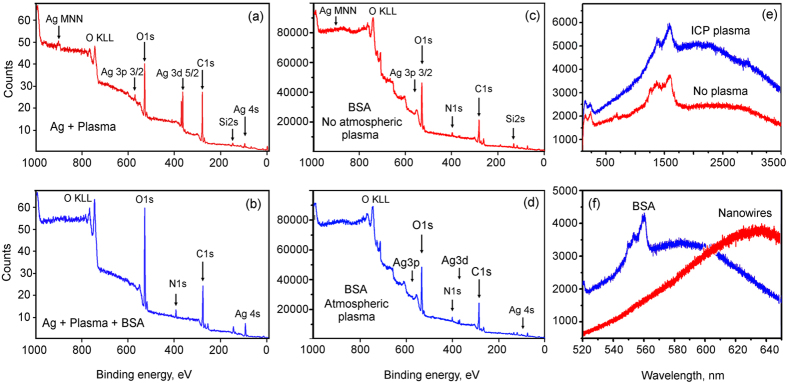
XPS and Raman characterization. (**a**,**b**) XPS spectra of the ICP-treated sample without BSA (**a**) and after incubation in BSA + water solution without the atmospheric plasma treatment. (**c**,**d**) XPS spectra of the ICP-treated samples after incubation in BSA + PBS solution, washing and drying, without (**c**) and with (**d**) atmospheric plasma treatment before BSA incubation. (**e**) Raman spectra of the samples with- and without ICP treatment, after incubation in the BSA + PBS solution, washing and drying. (**f**) Comparison of the Raman spectra for the Ag nanowire array with and without incubation in the BSA + PBS solution.

Firstly, the elemental compositions of all samples were characterized using an X-ray photoelectron spectroscopy (XPS) spectrometer (Specs-XPS, mode XP-50 high performance twin anode with focus 500 ellipsoidal crystal monochromator and PHOIBOS 150 MCD-9 analyzer). The binding energies of photoelectrons were measured in a range of 50–1000 eV. Several scans were made to maintain a high signal-to-noise ratio. The spectra taken from the ICP plasma-treated samples (without atmospheric jet plasma treatment) prior and after incubation in the water protein solution are shown in Fig. 6a,b (wide scan). Comparing these two spectra one can notice (i) a much stronger oxygen peak at 532 eV for the protein-incubated sample; (ii) the appearance of a nitrogen peak at 400 eV in the spectra of protein-incubated sample; (iii) a much stronger oxygen peak at 750 eV on the spectra of protein-incubated sample.

The spectra taken from the ICP plasma-treated samples incubated in the BSA + PBS solution (washed and dried as described above) with and without treatment by the atmospheric-pressure plasma jet (APPJ) are shown in Fig. 6(c,d), respectively. The nitrogen peaks are visible on both spectra, with the APPJ-treated sample featuring a stronger presence of nitrogen with respect to the oxygen peak in the same spectra.

The Micro-Raman characterization was performed using a Renishaw *inVia* spectrometer with laser excitations of 514 and 633 nm at a spot size of ~1 μm^2^. Raman spectra from multiple spots were collected to perform the average statistical analysis of the samples. The results of the Micro-Raman characterization are shown in Fig. 6(e,f). Figure 6(e) shows the Raman spectra collected from the array of Ag nanowires with and without the ICP treatment, respectively.

From these spectra one can conclude that the ICP plasma treatment resulted in a stronger resonance due to the changes in the entire structure. Indeed, shorter nanowires feature more ordered structure (as compared with the entangled long nanowires) which excites stronger signal by the interaction with a continuous supporting gold layer. Moreover, the APPJ plasma treatment apparently removed contaminations from the surfaces of the nanowires, and this resulted in a much stronger signal enhancement (more Raman spectra can be found in the Supporting Information).

To better characterize the cleaning effect of the atmospheric-plasma treatment, we have conducted an additional experiment on the fabrication of pure silver nanowires and taking the Raman spectra from them before and after the plasma treatment (keeping the laser power and other parameters constant). The results suggest that the changes in the characteristic features related to C-C bonds in the 520–630 nm range indicate a significant reduction of the amount of carbon contaminations after the plasma treatment. The relevant spectra are shown in the Supporting Information.

Figure 6(f) shows the Raman spectra taken from the ICP-treated nanowire arrays as well as the arrays incubated in the BSA + PBS solution. A comparison with the spectra taken from the sample without proteins shows enhancement of the signal and a significant change in the shape of the spectra. Furthermore, strong carbon peaks at 550 cm^−1^ are the signature of the proteins attached to the platform. Interestingly, the spectral intensity of the signal from the plasma-treated sample is significantly stronger than from the untreated nanowire array. This may be attributed to (i) shorter and more ordered nanowires which ensure deeper penetration of the protein molecules into array, and (ii) activation of the nanowire surface ensuring better protein attachment.

**Figure 7:**
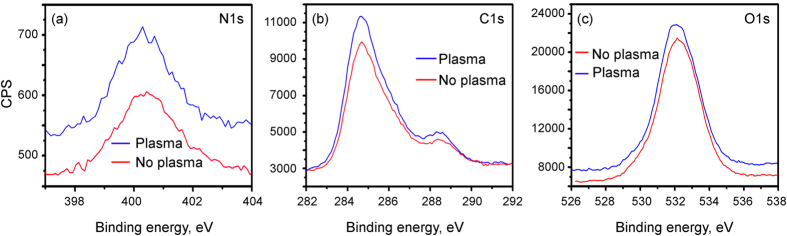
XPS spectra. XPS spectra (narrow scan) for N1s (**a**), C1s (**b**) and O1s (**d**) peaks. Nitrogen peak is stronger for the samples with plasma-treated Ag nanowires (**a**). The shoulders of the carbon C1s peak at 287 and 288 eV (**b**) correspond to carbon in C–O and C = O groups of the protein molecules. Oxygen peak is also stronger for the samples with plasma-treated Ag nanowires (**c**).

**Figure 8:**
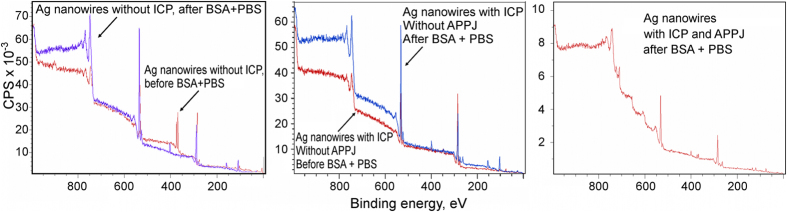
XPS spectra. XPS survey scans of the Ag nanowires, with and without atmospheric pressure plasma treatment, before and after incubation in BSA + PBS solution.

To compare the behavior of proteins on the APPJ-treated and non-treated arrays, we have performed narrow scans of the C1s and O1s peaks (see Fig. 7). To better analyze the structure, we have deconvoluted the peak of C1s spectra (see Supporting Information Fig. S2). The shoulders of the carbon C1s peak at 285.7 and 288.5 eV are signatures of the protein molecules containing carbon in C-O/N and O-C = O groups. Importantly, the full-width-at-half-maximum (FWHM) of these shoulders slightly narrowed for the plasma-treated nanowire samples, suggesting that more BSA molecules may be immobilized on the local surface area of XPS probe. C1s XPS signals of the Ag nanowires, with and without atmospheric pressure plasma treatment, before and after incubation in BSA + PBS solution are also shown on Fig. 8. The results of the curve fitting of the C1s XPS peaks of the Ag nanowires, with and without atmospheric pressure plasma treatment, before and after incubation in BSA + PBS solution is shown on the Supporting Information Fig. S3.

The oxygen O1s peak is also stronger for the APPJ-treated nanowire sample as compared to the one without APPJ treatment, as seen from the wide scans with respect to the N peak (Supporting Information Fig. S4). The XPS analysis demonstrates that the plasma treatment changes the binding energy between different elements and silver nanowires. It is particularly clear from the analysis of the C1s peak of BSA attached to plain SiO_2_ wafer, silver nanowires treated by the plasma, and silver nanowires without the plasma treatment (Supporting Information Fig. S5). Moreover, the oxygen-carbon binding energy and peak position also changed.

It should be stressed that the process of protein attachment to the plasma-treated nanostructured platforms is a complex process involving various possible chemical and physical routes. These specific mechanisms and routes of protein immobilization on our structure is essentially out of scope of this paper aimed at demonstration of the potential of using plasma-activated nanostructured metallic platforms for protein-related application. In our further studies we will try to study the details of proteins immobilization on similar platforms, thus ensuring the possibilities for optimization and enhancing this approach. The kinetics of the protein absorption on nanostructured platform is another very interesting and important question to be studied in detail in the future work.

## Conclusion

This work has reported on the fabrication and testing of the alumina membrane-based silver nanowire hierarchical bi-dimensional nanomaterial capable of trapping and retention of proteins. The dense arrays of gold-supported silver nanowires of about 100 nm in diameter were grown directly in the channels of nanoporous aluminium oxide membrane. After dissolving the alumina membrane, an hierarchical bi-dimensional nanomaterial was fabricated and tested by incubation in BSA protein. Additional treatment of the silver nanowires using low-temperature plasmas in the inductively-coupled plasma reactor have demonstrated that the morphology of the nanowire array can be controlled and the amount of the retained protein may be increased due to the plasma-related effects. A combination of the neutral gold sublayer with the antimicrobial properties of silver nanowires could significantly enhance efficiency of the biocatalytical platforms used in various biotechnological processes. The results may be important for designing novel nanomaterials for biotechnological and medical applications.

## Methods

### Fabrication of the nanoporous free-standing alumina membrane

To produce the nanoporous free-standing alumina membrane (to be used as a base matrix for the hierarchical structure), we have used a two-step anodization in an electrochemical anodization cell. Prior to fabrication of the membrane, aluminium was thoroughly cleaned in ultrasonic bath for 30 minutes using high-purity (99.5%) ethanol. Then, a standard voltage-reductional sequence process at a voltage of 24 V DC in oxalic acid (0.3 M) solution as electrolyte at the temperature of 0 °C was used (Fig. 1(b)) with a thin (250 μm) high-purity (99.999%) aluminium foil as a precursor material. A lead (99.5%) plate was used as a cathode electrode during the anodization. The durations of the first and second anodization processes were 1 and 8 hours, respectively. After the second anodization the residual aluminium was dissolved by dipping into mixture of CuCl_2_ (5%) and water solution of HCl (1:1) for several minutes. Then the fabricated membrane was dipped into the 5% H_3_PO_4_ for one hour to remove the barrier layer. The final thickness of the ready membrane was 20 μm. More details on the anodization process can be found elsewhere^58^.

### Fabrication of the nanowires array on gold layer

The prepared nanoporous membranes (external diameter of about 20 mm) were then coated with gold from one side. To achieve this, an AJA sputter coating system was used, and 300 nm Au layer was applied at 1.5 × 10^−2^ Torr and the coating rate of 0.2 nm × s^−1^. This layer was then used as an electrode during electrodeposition of the silver nanowires. After alumina dissolution this layer was used as a ‘supporting’ layer for the array of silver nanowires. Schematic of the alumina membrane with the gold layer is shown in Fig. 2(a). The silver nanowire array was grown in a three-electrode electrochemical cell of the Biologic VSP300 system. The membrane, an Ag/AgCl electrode, and a Pt plate were used as the working, reference, and counter electrodes of the cell, respectively. The silver nanowires arrays were prepared in 0.01 M AgNO_3_ aqueous electrolyte under cyclic voltammetry conditions by sweeping the potential from 0.0 to −0.6 V at room temperature (see the cyclic voltammogram in the Supporting Information provided), with the voltage change step of 5 m V × s^−1^. The experiment was stopped after 40 cycles. Afterwards, the membranes with Ag nanowires grown in the channels (Fig. 2) were washed out and dried with dry nitrogen jet.

### Atmospheric pressure plasma jet treatment

The atmospheric pressure plasma jet setup used to process the nanoporous membranes consists of a discharge microreactor which utilizes a tubular capillary to produce a plasma jet. Typically, the capillary is made of an insulating material such as quartz. A couple of electrodes are attached to the capillary, one electrode being grounded and the other one used to supply RF power at 375 kHz, 40 W to the discharge. A ground electrode was made in the form of a ring installed over the tubular capillary near the gas exit, and the powered electrode was made in the form of a thin, long metallic wire and installed inside the tubular capillary. A limiting resistor of several Ohms was also used to restrict the total discharge current in case of a breakdown which could result in arcing. A standard gas supply system including bottles and mass flow controller was used to maintain the desired flow rate of up to 500 sccm for He in the discharge. The total treatment time was 2 min for each sample. More information about the APPJ processing can be found elsewhere^54^,^55^.

## Additional Information

**How to cite this article**: Fang, J. *et al.* Protein retention on plasma-treated hierarchical nanoscale gold-silver platform. *Sci. Rep.* **5**, 13379; doi: 10.1038/srep13379 (2015).

## Supplementary Material

Supplementary Information
