# Supplementary material for: Protein retention on plasma-treated hierarchical nanoscale gold-silver platform
Source: Sci Rep. 2015 Aug 26;5:13379. doi: 10.1038/srep13379 (PMC4549625; doi:10.1038/srep13379)
Supplement: Supplementary Information [file srep13379-s1.pdf]

Supplementary Information

Protein retention on plasma-treated hierarchical nanoscale gold-silver platform

Jinghua Fang[1,2], Igor Levchenko[1,3,], Anne Mai-Prochnow[1], Michael Keidar[4], Uros Cvelbar[5], Gregor Filipic[5], Zhaojun Han[1], and Kostya (Ken) Ostrikov[1,3,6]*

[1] Plasma Nanoscience Laboratories, Manufacturing Flagship, Commonwealth Scientific and Industrial Research Organisation (CSIRO), P.O. Box 218, Lindfield, NSW 2070, Australia.

[2] School of Physics, University of Melbourne, Parkville, VIC, Australia, 3010.

[3] Plasma Nanoscience, School of Physics, The University of Sydney, Sydney, NSW 2006, Australia.

[4] Department of Mechanical and Aerospace Engineering, The George Washington University, Washington, DC 20052, USA

[5] Jozef Stefan Institute, Dep. of Surface Eng. and Optoelectronics, Jamova 39, 1000 Ljubljana, Slovenia, EU

[6]School of Chemistry, Physics, and Mechanical Engineering, Queensland University of Technology, Brisbane, QLD 4000, Australia.

*E-mail: I.Levchenko@post.com

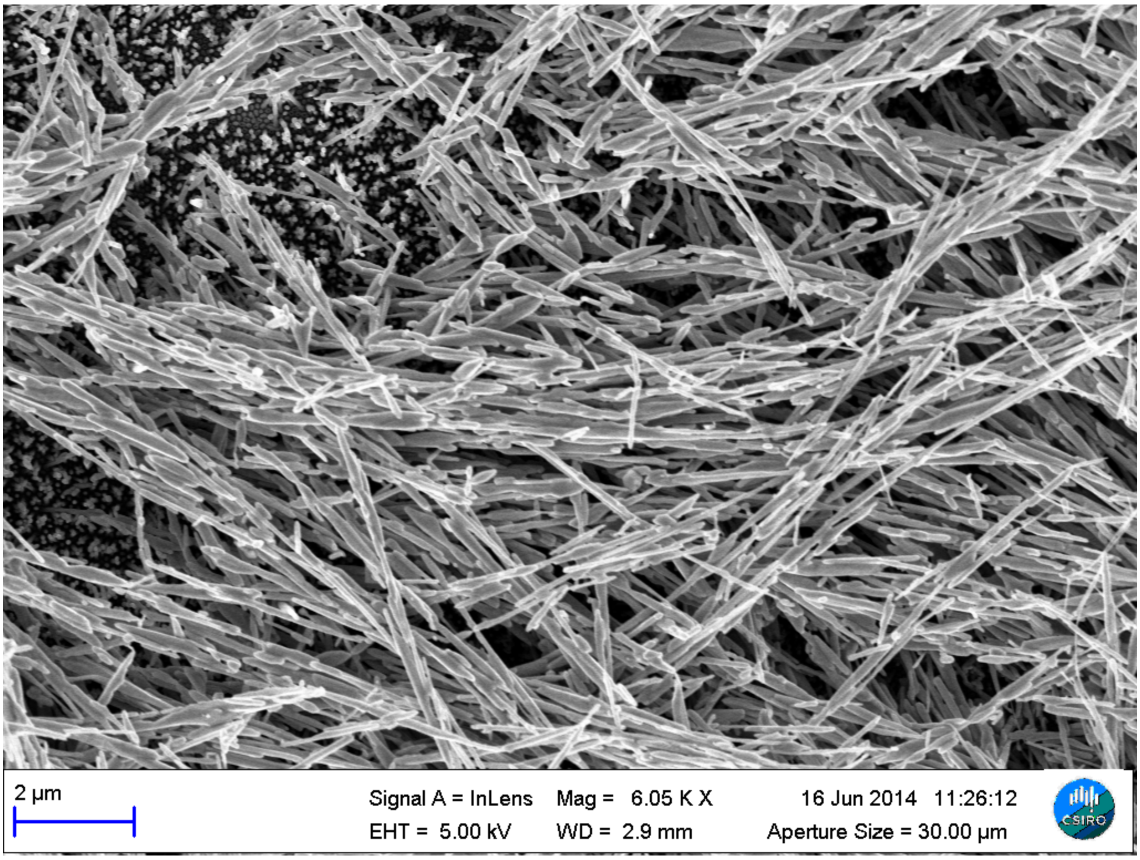

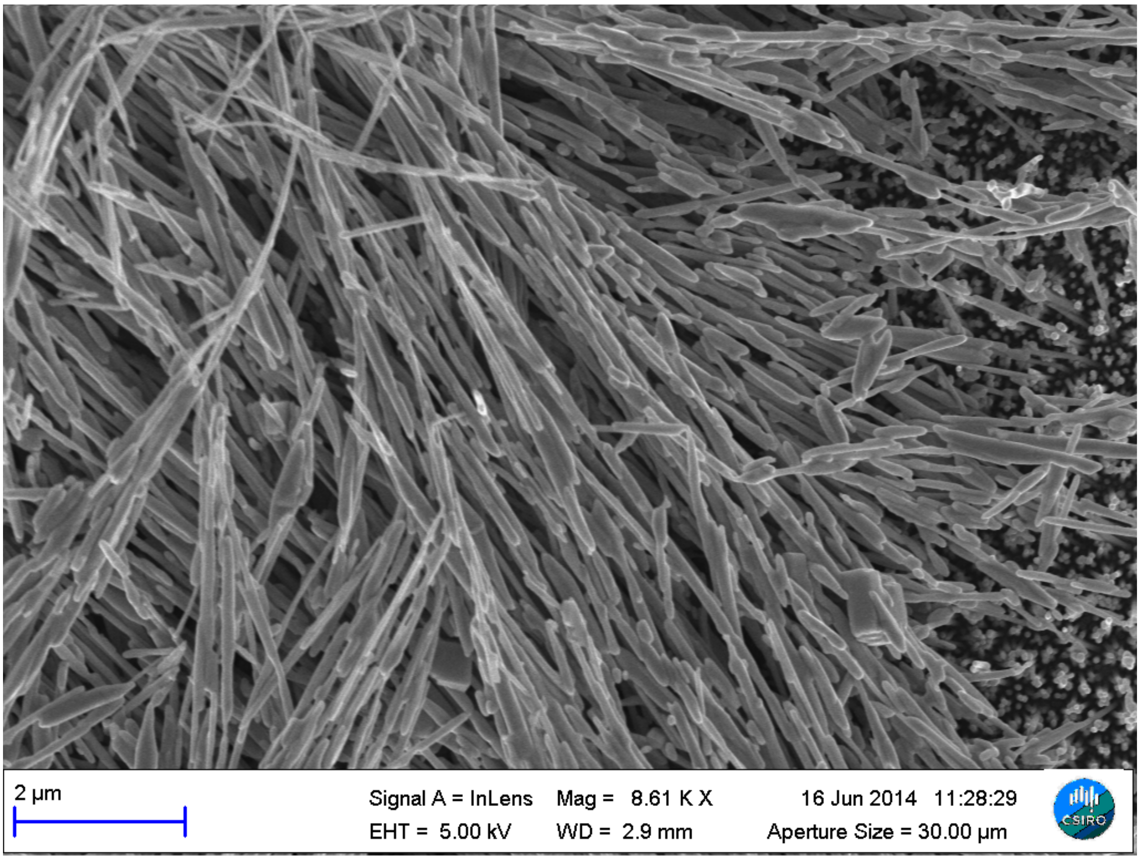

Figure S1 | SEM images of silver nanowires on nanoporous membrane

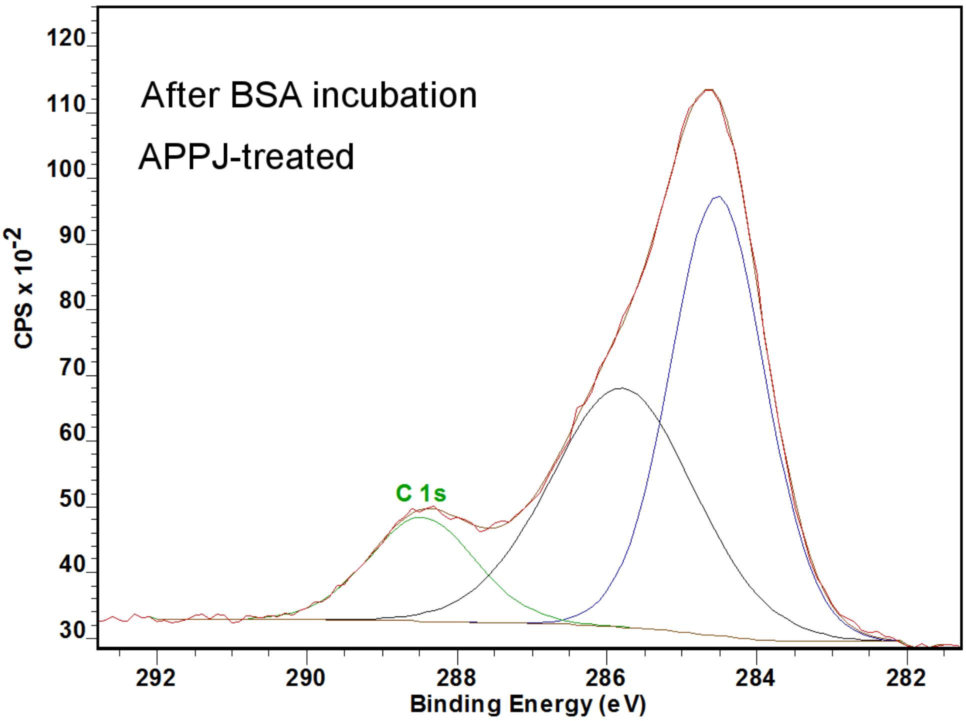

Figure S2 | Peak deconvolution of C 1s spectra XPS survey scan for APPJ-treated samples after BSA incubation: the three peaks at 284.5, 285.8, and 288.5 eV corresponds to C-C, C-O/N, and O-C=O bonds, with FWHM of 1.5, 2.2, and 1.7 eV respectively

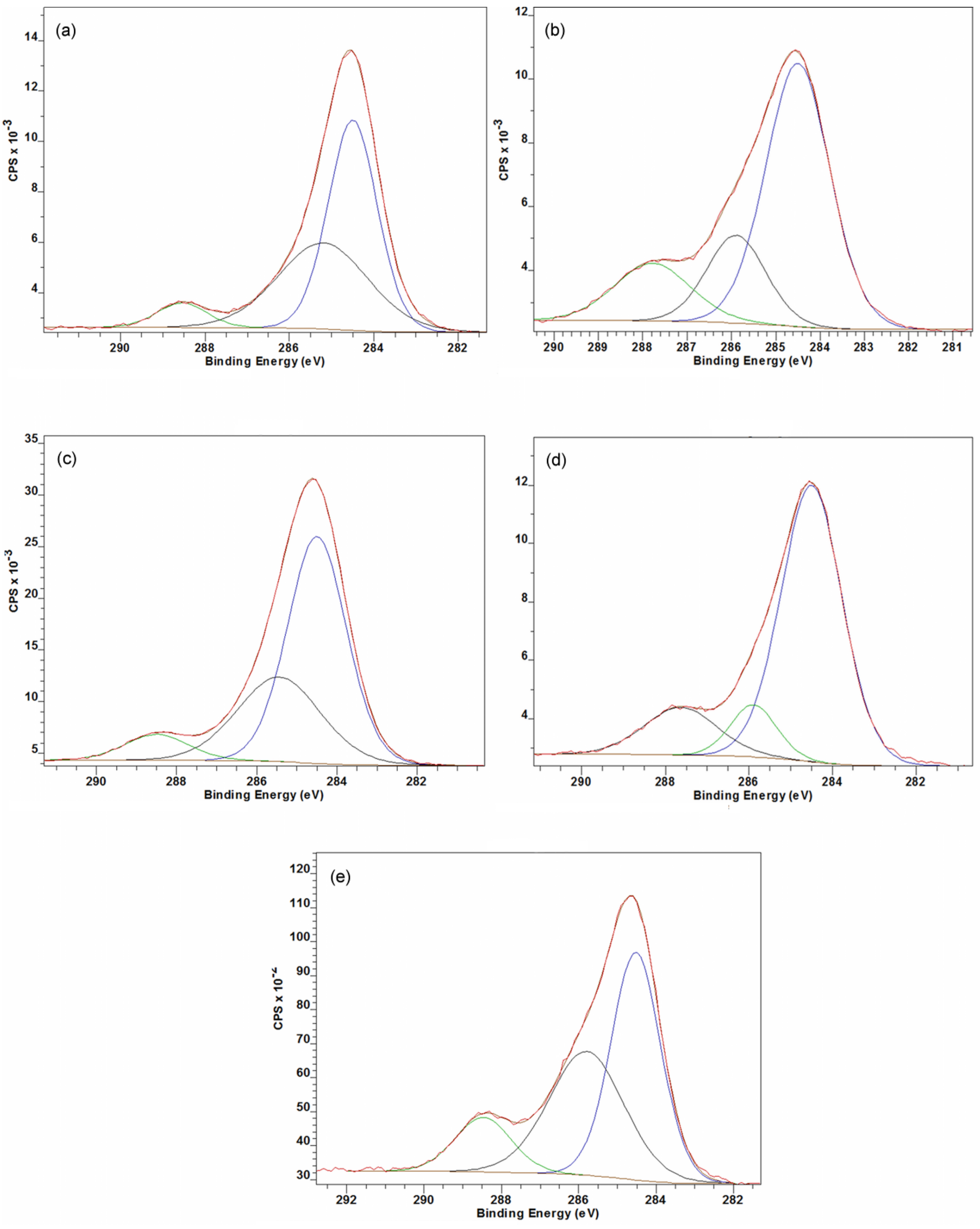

Figure S3 | Results of curve fitting of the C1s XPS peaks of the Ag nanowires. (a) C 1s spectrum for Ag nanowires without ICP treatment, before BSA+PBS; (b) C 1s spectrum of Ag nanowires without ICP treatment, after BSA+PBS; (c) C 1s spectrum of Ag nanowires with ICP but without APPJ treatment, before BSA+PBS; (d) C 1s spectrum for Ag nanowires with ICP but without APPJ treatment, after BSA+PBS; (e) C 1s spectrum for Ag nanowires with ICP and APPJ treatments, after BSA_PBS.

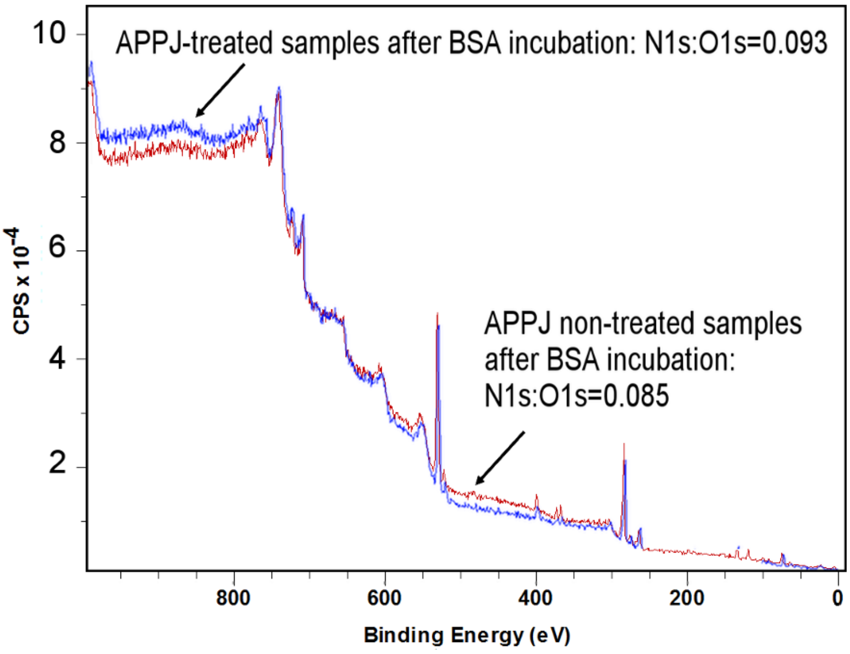

Figure S4 | XPS survey scans for the APPJ-treated and APPJ non-treated samples after BSA incubation

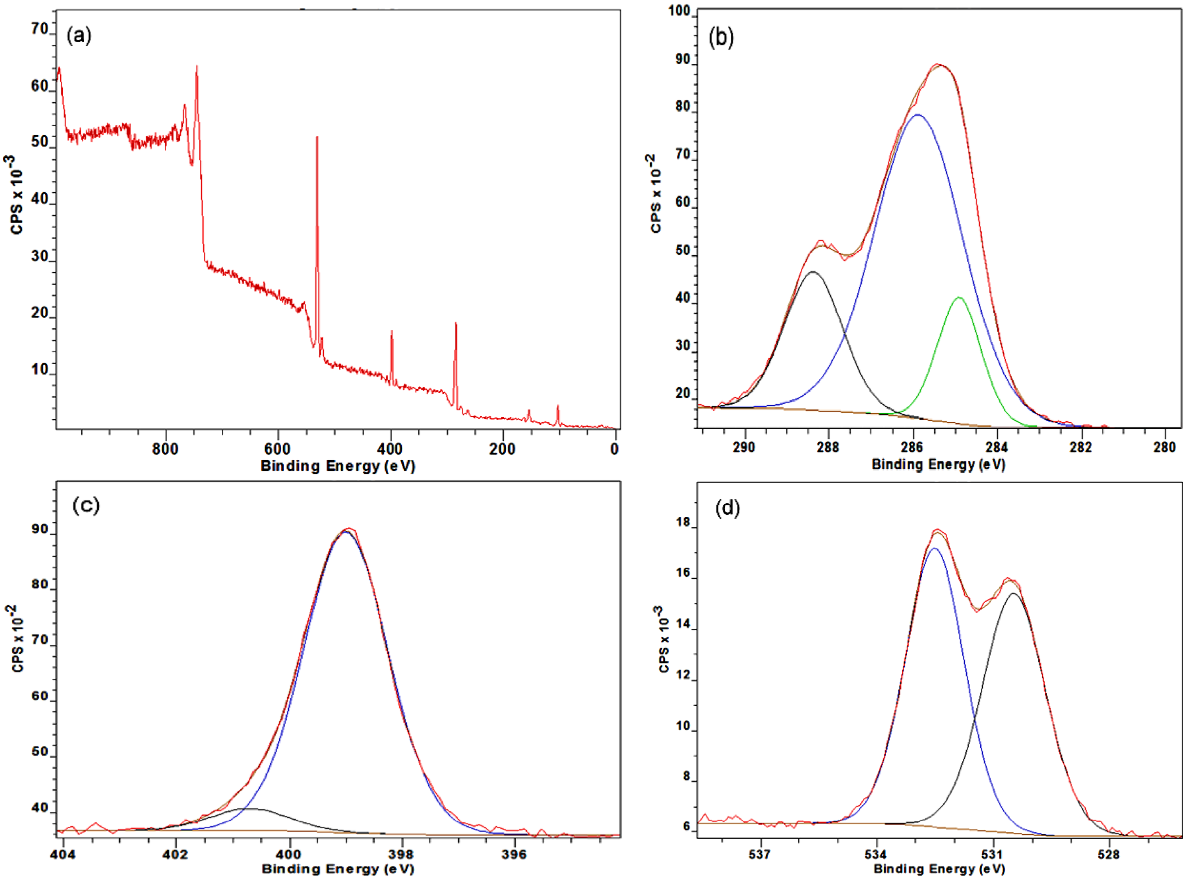

Figure S5 | XPS analysis of BSA on Si. (a) XPS survey scan of BSA on silicon; (b, c, d) C 1s, N 1s and O 1s spectra of BSA on silicon.

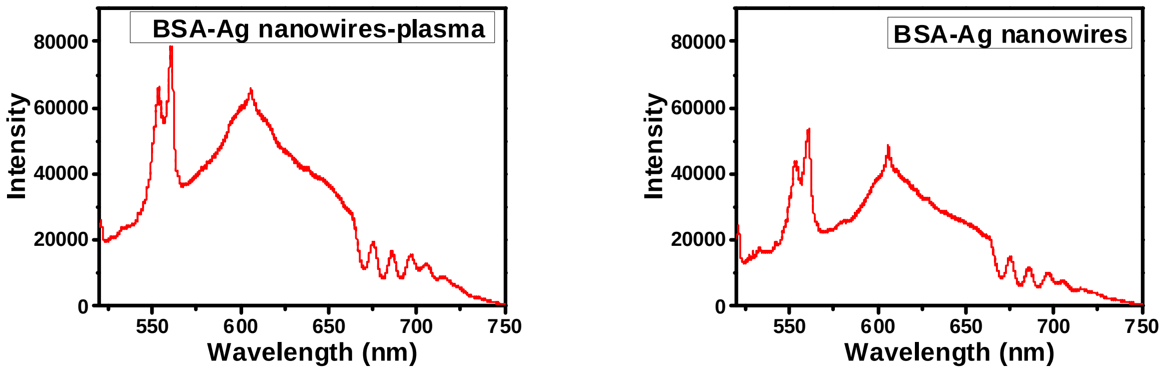

Figure S6 | Raman spectra taken from the samples treated (a) and not treated (b) with atmospheric plasmas (a). The signal is apparently stronger on samples treated with the plasma.

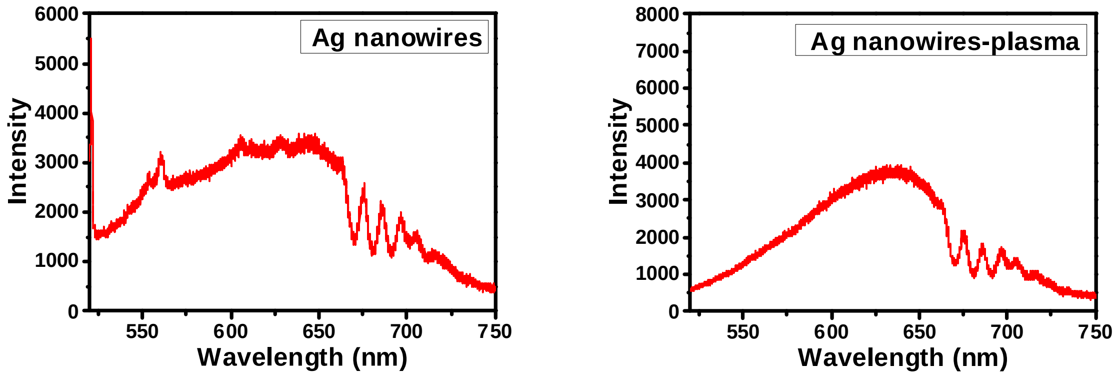

Figure S7 | Raman spectra taken from the silver nanowire samples not treated (a) and treated (b) with atmospheric plasmas (a). Changes in the characteristic features related to C-C bonds in the 520 – 630 nm range suggest a significant reduction of the amount of carbon contaminations after the plasma treatment.

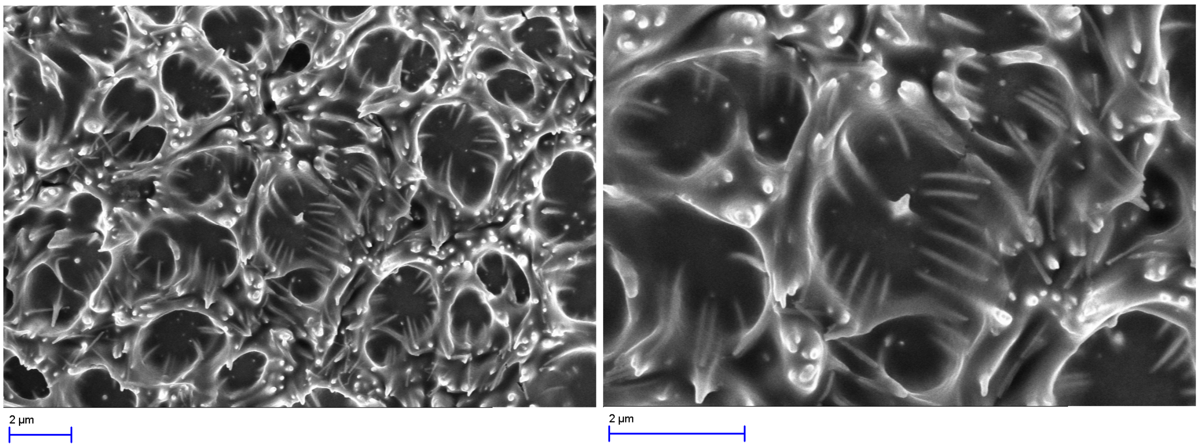

Figure S8 | SEM images taken from the silver nanowire samples incubated in proteins. Changes in the array morphology demonstrates presence of organic material.
